# Development and validation of a simple questionnaire for the identification of hereditary breast cancer in primary care

**DOI:** 10.1186/1471-2407-9-283

**Published:** 2009-08-14

**Authors:** Patricia Ashton-Prolla, Juliana Giacomazzi, Aishameriane V Schmidt, Fernanda L Roth, Edenir I Palmero, Luciane Kalakun, Ernestina S Aguiar, Susana M Moreira, Erica Batassini, Vanessa Belo-Reyes, Lavinia Schuler-Faccini, Roberto Giugliani, Maira Caleffi, Suzi Alves Camey

**Affiliations:** 1 Programa de Pós-Graduação em Genética e Biologia Molecular, Universidade Federal do Rio Grande do Sul (UFRGS), Porto Alegre, Brazil; 2 Laboratório de Medicina Genômica, Hospital de Clínicas de Porto Alegre (HCPA), Porto Alegre, Brazil; 3 Núcleo Mama Porto Alegre, Associação Hospitalar Moinhos de Vento, Porto Alegre, Brazil; 4 Serviço de Genética Médica, HCPA, Porto Alegre, Brazil; 5 Departamento de Genética, UFRGS, Porto Alegre, Brazil; 6 Programa de Pós-Graduação em Medicina: Ciências Médicas, UFRGS, Porto Alegre, Brazil; 7 INAGEMP, Instituto Nacional de Genética Médica Populacional, Porto Alegre, Brazil; 8 Bolsista PROPESQ, UFRGS, Porto Alegre, Brazil; 9 Programa de Pós-Graduação em Epidemiologia, UFRGS, Porto Alegre, Brazil; 10 Departamento de Estatística, Instituto de Matemática, UFRGS, Porto Alegre, Brazil

## Abstract

**Background:**

Breast cancer is a significant public health problem worldwide and the development of tools to identify individuals at-risk for hereditary breast cancer syndromes, where specific interventions can be proposed to reduce risk, has become increasingly relevant. A previous study in Southern Brazil has shown that a family history suggestive of these syndromes may be prevalent at the primary care level. Development of a simple and sensitive instrument, easily applicable in primary care units, would be particularly helpful in underserved communities in which identification and referral of high-risk individuals is difficult.

**Methods:**

A simple 7-question instrument about family history of breast, ovarian and colorectal cancer, FHS-7, was developed to screen for individuals with an increased risk for hereditary breast cancer syndromes. FHS-7 was applied to 9218 women during routine visits to primary care units in Southern Brazil. Two consecutive samples of 885 women and 910 women who answered positively to at least one question and negatively to all questions were included, respectively. The sensitivity, specificity and positive and negative predictive values were determined.

**Results:**

Of the 885 women reporting a positive family history, 211 (23.8%; CI95%: 21.5–26.2) had a pedigree suggestive of a hereditary breast and/or breast and colorectal cancer syndrome. Using as cut point one positive answer, the sensitivity and specificity of the instrument were 87.6% and 56.4%, respectively. Concordance between answers in two different applications was given by a intra-class correlation (ICC) of 0.84 for at least one positive answer. Temporal stability of the instrument was adequate (ICC = 0.65).

**Conclusion:**

A simple instrument for the identification of the most common hereditary breast cancer syndrome phenotypes, showing good specificity and temporal stability was developed and could be used as a screening tool in primary care to refer at-risk individuals for genetic evaluations.

## Background

According to the World Cancer Report, cancer rates will increase by 50% from 10 million new cases estimated for the year 2000 to 15 million new cases per year in 2020. However, the report also provides evidence that efforts in the control of risk factors and public health strategies to increase surveillance and thus promote early cancer detection could prevent as many as one third of cancers diagnosed in the world [1,2].

Among women, breast cancer (BC) is the most prevalent malignant tumor, and one in four cancers diagnosed in women worldwide is a cancer of the female breast. More than 1.1 million women are diagnosed each year with the disease and its incidence rates are still increasing in many countries [2,3]. In Brazil, BC is a significant public health problem, due to its morbidity, and high incidence and mortality rates. About half of the affected women are diagnosed in advanced stages and not surprisingly, mortality rates are still increasing [4,5]. The State of Rio Grande do Sul (RS) has one of the highest BC incidence rates of the country with a predicted rate of 85.50 per 100,000 estimated for the year of 2008 – which is comparable to the USA and North Europe. The State's capital, Porto Alegre, has an even higher BC incidence rate, with 119.72 new cases per 100,000 women [4].

Positive family histories of BC and other tumors are associated with an increased risk for developing the disease and are recognized as indicators for the identification of high-risk, genetically predisposed individuals [6,7]. Overall, an estimated 5–10% of all breast cancers is hereditary, i.e., caused by a germline mutation in a predisposition gene that confers to its carrier a significantly higher cancer risk. Data on the prevalence of hereditary breast cancer in Brazil is scarce, and although founder mutations in cancer predisposition genes have been described in the country, there is no evidence for increased frequency of hereditary cancer syndromes in the Brazilian population [[8], Garritano S, Gemignani F, Palmero EI, Olivier M, Martel-Planche G, Calvez-Kelm FL, Brugières L, Vargas FR, Brentani RR, Ashton-Prolla P, Landi S, Tavtigian SV, Hainaut P, Achatz MI. High frequency of the cancer-predisposing *TP53 *mutation p.R337H in the population of Southern Brazil: evidence for a founder effect, submitted to *Human Mutation*]. Germline mutations in the *BRCA1 *and *BRCA2 *genes are related to an increased risk for breast, ovarian and other cancers in the autosomal dominant Hereditary Breast and Ovarian Cancer (HBOC) syndrome [9,10]. Clinically significant *BRCA *mutations are estimated to occur in 1 in 300 to 500 persons in the general population [11]. Other BC predisposition genes, such as *TP53 *(associated with Li-Fraumeni and Li-Fraumeni-*like *syndromes, LFS/LFL), *PTEN *(associated with Cowden syndrome, CS) and *CHEK2 *(associated with Hereditary Breast and Colorectal Cancer, HBCC) have been identified and are thought to have important, albeit lower contributions to hereditary breast cancer (HBC) [12-17]. Specific features in the family history may suggest the diagnosis of a hereditary breast cancer syndrome (HBCS) (i.e. pre-menopausal BC, male BC, bilateral BC and family history of BC and ovarian cancer – OC) [9,11].

Taking a family history has long been considered an integral part of the medical evaluation and is particularly important in genetic risk evaluation and risk management protocols, where it may ultimately help in the design of strategies to reduce cancer-associated mortality [18]. Health care providers have a professional and legal duty to obtain sufficient family history information to perform adequate cancer risk assessments [19,20]. However, despite its importance, information about the family history is not routinely or sufficiently collected outside the setting of cancer risk evaluation programs; in primary care, it has been described as a neglected area [21,22]. In addition, even among highly educated women, knowledge of HBC risk factors that could influence self-referral is usually scarce [23]

In Brazil, several barriers to the identification and counseling of individuals at-risk for HBCS have been identified and include lack of well established cancer genetic counseling services, absence of specific training programs in cancer genetics, small numbers of certified clinical geneticists and their unequal geographic distribution in the country [24]. The genetic cancer risk assessment process is an activity that requires specific training, in-depth knowledge of the subject, a significant amount of time and a multidisciplinary approach. Although little is known about the efficacy and the cost-benefit relationship of community-based programs of identification of individuals at-risk for hereditary cancer, training of primary health care professionals and use of simple tools to facilitate the identification of these individuals may be helpful to ensure proper referrals and to optimize evaluations, especially in low resource countries [24,25].

In this study, a simple instrument to inquire about the family history of BC, OC and CRC was developed to identify women at-risk for HBCS during their primary care visits. We tested the instrument's sensitivity and positive and negative predictive values to identify different HBC phenotypes and its ability to identify women with higher lifetime risks for developing BC as estimated by current risk models.

## Methods

### Development of an instrument to identify women at risk for HBC in primary care

In April of 2004, a population-based cohort study (the Núcleo Mama Porto Alegre – NMPOA Cohort) was started in 18 primary care units of the city of Porto Alegre, Southern Brazil [26,27]. The purpose was to collect demographic and epidemiologic data of a large sample of women and test a model for community-based BC screening in an underserved population that relies upon the *Programa Saúde da Família *(PSF or Family Health Program). This program was created in the mid-90's and is based upon multidisciplinary teams, composed by a physician, nurse, one or two nurse assistants and 4–6 lay community health workers. This team provides primary health care to a geographically defined group of approximately 600 families. The *Programa Saúde da Família *has expanded rapidly and nowadays provides health care to about half of the population [28-30].

To identify individuals and families at risk for HBC syndromes within the cohort, an instrument with seven questions, named FHS-7, was developed and applied to all women that visited the participating primary care units from April 2004 to March 2006 (Table 1). FHS-7 was originally designed to identify women at-risk for the HBOC and included questions on features that have been associated with an increased likelihood of clinically significant *BRCA *mutations [11]. In addition, a question about family history of BC and/or CRC was included due to a previous suggestion of a higher than expect prevalence of such association in cancer genetic clinics of Porto Alegre [24]. Patients older than 18 years answering positively to at least one of these questions were referred to genetic cancer risk assessment (GCRA) where interviews with clinical geneticists and detailed pedigree analyses were performed. Ethical approval was obtained from the institutional ethics committees and patient inclusion required signature of informed consent.

**Table 1:** The FHS-7 questionnaire: intra-class correlation (ICC) between responses to individual questions in two settings: primary care unit (PCU) and immediately before genetic cancer risk assessment (GCRA) and total of positive answers at GCRA interview.

#	Question	Positive answers	ICC (PCU/GCRA)
1	Did any of your 1^st ^degree relatives have breast *or *ovarian cancer?	348 (19.4%)	0.87
2	Did any of your relatives have bilateral breast cancer?	84 (4.7%)	0.56
3	Did any man in your family have breast cancer?	2 (0.1%)	0.33
4	Did any woman in your family have breast *and *ovarian cancer?	5 (0.3%)	0.08
5	Did any woman in your family have breast cancer before the age of 50 years?	537 (29.9%)	0.79
6	Do you have 2 or more relatives with breast *and/or *ovarian cancer?	221 (12.2%)	0.58
7	Do you have 2 or more relatives with breast *and/or *bowel cancer?	251 (14.0%)	0.53

### Patient recruitment and definition of study groups

**Figure 1:**
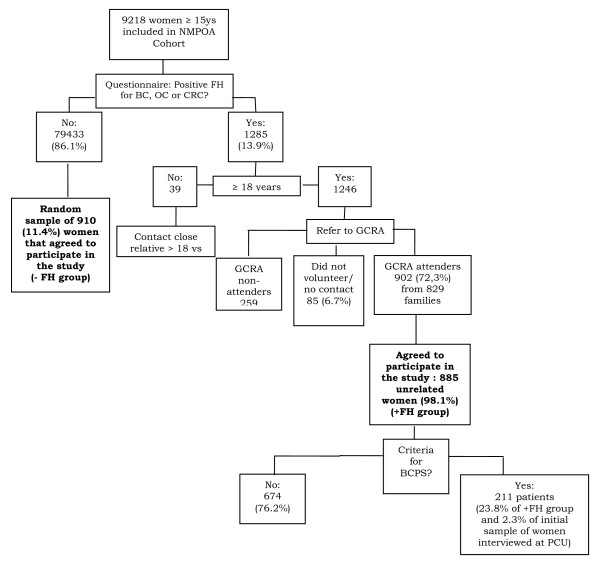
**Overview of the questionnaire validation procedure and its results**. NMPOA = Nucleo Mama Porto Alegre cohort; FH = family history; BC = breast cancer; OC = ovarian cancer; CRC = colorectal cancer; GCRA = genetic cancer risk assessment; BCPS = breast cancer predisposition syndrome; PCU = primary care unit.

Of the 9218 women enrolled in the NMPOA cohort, 1285 (13.9%) women who visited the primary care units participating in the cohort, answered positively to at least one of the seven questions about family history of cancer in FHS-7 and those above age 18 years (1246) were referred for GCRA. Of these, 902 women from 829 families were seen for GCRA during a period of three years (2005–2007), and 885 agreed to participate in the study presented here (Figure 1). Study subjects included: (a) 885 unrelated women with a positive family history of cancer and (b) 910 unrelated women of the same cohort who denied a family history of cancer. Women in these two groups answered FHS-7 in their primary care units and confirmed the family history during a genetic evaluation session in a specialized health care center (NMPOA). In addition, a separate random sample (n = 171) of women participating in the cohort completed FHS-7 twice (at recruitment in the primary care unit and during their mammographic screening visit at the same specialized health care center, NMPOA). The random selection was done among those individuals whose time interval between the first interview at the primary care units and second interview during routine mammographic screening was less than 30 days.

### Genetic Cancer Risk Assessment (GCRA)

Genetic evaluation included medical and family histories recorded in detailed pedigrees with information traced as far backwards and laterally as possible, extending to paternal lines and including a minimum of three generations using standard methods. Confirmation of the cancer family history was attempted in all cases and pathology reports, medical records and/or death certificates were obtained whenever possible.

**a) Lifetime breast cancer risk estimates (LBCRE)**: were obtained using the Claus Tables and the Gail and Tyrer-Cuzick models [31-33]. Upper age limit considered for the three LBCRE is 79, 90 and 80 years, respectively.

**b) Patients at high-risk for a hereditary breast cancer syndrome (HBC)**: all patients fulfilling criteria for a BC predisposition syndrome upon pedigree analysis were classified in this group. Criteria for the definition of HBC syndromes are detailed below. For the HBOC syndromes, the American Society of Clinical Oncology (ASCO) criteria were used [35,36]. In addition, prior probabilities of carrying a *BRCA1 *or *BRCA2 *mutation were determined for each patient using mutation prevalence tables and the Penn II mutation prediction model [35-37]. All pedigrees were reviewed by at least two clinical geneticists to assess presence of criteria for Li-Frameni syndrome, Li-Fraumeni-Like syndrome, HBCC or other cancer predisposition syndromes. For Li-Fraumeni syndrome, the original criteria described by Li and Fraumeni [12] were used; for Li-Fraumeni-Like syndrome, pedigrees were classified according to the criteria of Birch [13] and Eeles [14] and for HBCC, the Meijers-Heijboer and Nasseem criteria were used [15-38].

### Statistical Analysis

Intra-class correlation (ICC) were used to verify the agreement between results obtained by the instrument and the genetic cancer risk evaluation. In addition, ICC was used to measure agreement between answers to the instrument when applied in two different occasions, for temporal stability verification.

To determine the cutoff value for the original FHS-7 instrument, the ROC curve was used and the values for specificity and sensitivity were calculated. To compare the mean risk estimates obtained using the Gail, Claus and Tyrer-Cuzick models, t test for different samples was applied. In all analyses a significance level of 0.05 was considered and two-sided analyses were performed.

## Results

Demographic and reproductive data of the sample studied are summarized in Table 2. The mean age at assessment was 47.7 years (SD = 11.7); the majority (73.5%) of women enrolled had up to eight years of education and the average number of years of education was 6.3 (SD = 3.2). Risk estimates were obtained for all women and the mean estimates for the entire group were 8.14% (CI95%: 8.07–8.21), 8.91% (CI95%: 8.85–8.98) and 7.02% (CI95%: 6.94–7.09) using the Gail, Claus and Tyrer-Cuzick models, respectively.

**Table 2:** Demographics and variables of the women enrolled in this study.

	Women reporting a FH^1 ^of cancer in PCU^2 ^(n = 885)		Women without FH^1 ^report of cancer in PCU^2 ^(n = 910)	
	
	N(%)	Mean(SD)	N(%)	Mean(SD)
**Age at assessment**	-	43.9 (12.7)	-	51.4 (9.2)
**BMI**				
≤18.5	5 (0.6)	-	12 (1.3)	-
18.51–25	293 (33.6)	-	232 (25.5)	-
25.01–30	291 (33.3)	-	362 (39.8)	-
>30	284 (32.5)	-	304 (33.4)	-
**Age at menarche**	-	12.7 (1.7)	-	13.0 (1.7)
**Parity**				
No children	107 (12.2)	-	65 (92.7)	-
One or more children	777 (87.8)	-	844 (7.2)	-
**Age at birth of first child**	-	21.5 (5.0)	-	21.7 (5.2)
**Reproductive Status**				
Pre-menopausal	574 (64.9)	-	448 (50.3)	-
Post-menopausal	311 (35.1)	-	458 (49.7)	-
**Age at menopause**	-	47.0 (5.4)	-	46.8 (5.5)
**Previous biopsy**	78 (8.8)	-	66 (7.3)	-
**Hormone replacement therapy**	72 (8.1)	-	99 (10.9)	-
**Consanguinity**^‡^	64 (7.4)	-	53 (5.8)	-

^1 ^FH = Family History.

^2 ^PCU = Primary Care Unit.

(‡) Evidence of consanguinity within family, regardless of relationship to the proband.

After application of FHS-7, 885 women responded affirmatively to at least one question: 375 women (42.4%) reported a first-degree family history of BC or OC, 109 (12.3%), bilateral BC, 10 (1.1%) male BC, 40 (4.5%) BC and OC double primary tumors, 557 (62.9%), BC under the age of 50 years, 222 (25.1%), ≥ 2 relatives with BC and/or OC and 222 (26.2%) ≥ 2 relatives with BC and/or CRC in their families.

Table 3 shows that there was strong agreement between answers to questions 1 and 5 and their corresponding family history after GCRA. Agreement was moderate between the answers to questions 2, 6 and 7 obtained in the primary care units and the corresponding family history obtained during the genetic evaluation. The ICC for the question 3 was weak and for the question 4 was immeasurable; and this was likely due to small number of the positive answers.

**Table 3:** Total number of women fulfilling different criteria for hereditary breast cancer syndromes in both study groups.

	Women reporting a FH^1 ^of cancer in PCU^2 ^(n = 885)		Women with a negative FH^1 ^for cancer in PCU^2 ^(n = 910)	
	n	%	n	%
ASCO criteria for HBOC syndrome^3^	64	7.2	2	0.2
High risk HBOC syndrome^4^	75	8.5	2	0.2
HBCC criteria^3^	26	2.9	0	0.0
LFL syndrome^3^	141	16.0	29	3.2
HBC syndrome (overall)^5^	211	23.8	30	3.3

^1 ^FH = Family History.

^2 ^PCU = Primary Care Unit.

^3 ^Criteria as described in materials and methods.

^4 ^ASCO criteria and/or prior probability of a BRCA mutation ≥ 30% using Myriad mutation prevalence tables and the Penn II mutation prediction model.

^5 ^Includes Hereditary Breast and Ovarian Cancer (HBOC), Hereditary Breast and Colorectal Cancer (HBCC) and Li-Fraumeni-*like *syndrome (LFL) criteria.

Regarding temporal stability of the instrument, ICC for each of the 7 questions were: 0.72 for BC or OC in a first-degree relative, 0.74 for BC diagnosed <50 years in a relative, 0.61 for ≥ 2 relatives with BC and/or OC, 0.38 for bilateral BC in a relative, 0.17 multiple BC and OC in a relative and 0.47 for ≥ 2 relatives with BC and/or CRC in the family. For the question about multiple BC and OC in a relative there were only 5 positive answers in GCRA. For the male BC question there was no positive answer and thus, the ICC coefficient could not be calculated. When considering the instrument as a whole, the presence of at least one positive answer in both instrument applications had an ICC of 0.65.

**Figure 2:**
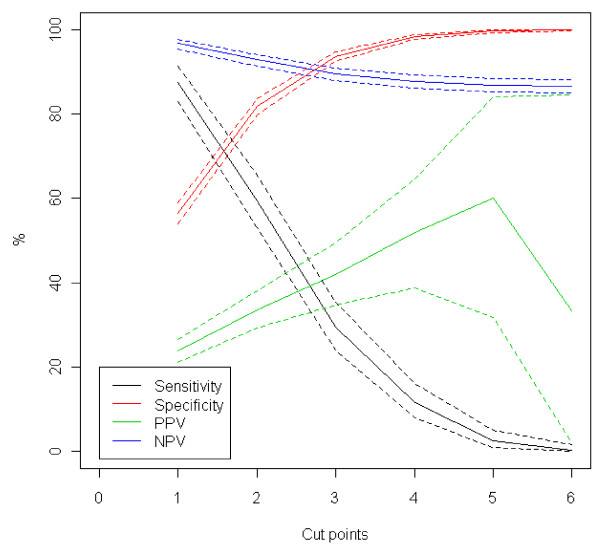
**Specificity, sensitivity, positive and negative predictive values and their respective 95% confidence intervals, for each of different cut points used for the identification of the hereditary breast cancer phenotype**. PPV = positive predictive value; NPV = negative predictive value.

Application of FHS-7 in primary care units to screen for HBC phenotypes resulted in the identification of 211/885 individuals (23.8%; CI95%: 21.5–26.2) with a family history fulfilling such diagnoses, including the HBOC, HBCC and Li-Fraumeni-Like syndromes; no patient reported a family history consistent with Li-Fraumeni syndrome. In contrast, only 30/910 (3.3%; CI95%: 2.9–3.7) individuals that responded negatively to all of the 7 questions of the instrument had criteria for one or more of such phenotypes upon GCRA (Table 3). Overall, in a primary care setting, using weighted estimation, 6.2% (IC95%: 5.7–6.6) of women had a phenotype of one of the HBCS. Figure 2 show that the best cut point is 1, since it has the highest sensitivity and negative predictive value. In addition, the positive predictive value decreases when we consider 6 as cut point. This is unexpected, but occurs because there is no case with six or more affirmative answers. Table 4 describes the ability of FHS-7 to identify women with different clinical criteria for HBCS by family history using a cut point = 1.

**Table 4:** Ability of the FHS-7 to identify women with clinical criteria (family history) for hereditary breast cancer syndromes (n = 1795).

Criteria	AUC (CI 95%)	Cut point = 1			
		
		Sensitivity (CI 95%)	Specificity (CI 95%)	**PPV**^1^**(CI 95%)**	**NPV**^2^**(CI 95%)**
ASCO criteria^3^	0.85 (0.81–0.89)	97.0 (91–100)	52.0 (50–55)	7.0 (6–9)	100.0 (99–100)

High risk HBOC syndrome^4^	0.86 (0.82–0.90)	97.0 (92–100)	53.0 (50–55)	8.0 (7–10)	100.0 (99–100)

HBCC criteria^3^	0.86 (0.84–0.88)	100.0 (89–100)	51.0 (49–54)	3.0 (2–4)	100.0

LFL syndrome^3^	0.72 (0.68–0.76)	83.0 (77–88)	54.0 (52–56)	16.0 (14–18)	97.0 (96–98)

HBC syndrome (overall)^5^	0.83 (0.81–0.85)	88.0 (83–91)	56.0 (54–59)	24.0 (21–27)	97.0 (95–98)

^1 ^PPV = positive predictive value.

^2 ^NPV = negative predictive value.

^3 ^Criteria as described in materials and methods.

^4 ^ASCO criteria and/or estimated prior probability of a BRCA mutation ≥30%.

^5 ^Includes Hereditary Breast and Ovarian Cancer (HBOC), Hereditary Breast and Colorectal Cancer (HBCC) and Li-Fraumeni-*like *syndrome (LFL) criteria.

## Discussion

Although the family history of cancer is the single most important tool for the initial identification of hereditary cancer syndromes and usually does not require any sophisticated technologies, little attention and time is usually spent to obtain a detailed pedigree in routine clinical practice. Even in tertiary health care institutions, recording of a comprehensive pedigree is uncommon. Most health care professionals outside clinical genetics are not trained to obtain a detailed family history, usually do not have sufficient time and do not recognize the power of this simple tool for disease identification and prevention [39]. In fact, reported family history of cancer, especially when in first-degree relatives appears to be quite reliable, and such reliability may exist regardless of educational level of the informant [21]. Since most at-risk families are identified *first *by a primary health care professional, development of simple tools to facilitate the identification of such families at the primary care level can be useful and effective to optimize referrals [40,41], especially in low resource countries. In this study, we developed and validated a simple family history questionnaire, FHS-7, for application at the primary care level and used a cut point of at least one positive question to determine referral for genetic evaluation by a trained specialist.

Overall, in the process of instrument validation, most of the women responding positively to at least one of the seven questions at the primary care units had also at least one positive finding regarding the same seven family history features identified during GCRA by a trained clinical geneticist. This indicates that there is a good agreement between the information obtained with the instrument in primary care and the information obtained during GCRA. For some of the questions, such as those inquiring about a family history of male BC and multiple primary (breast and ovarian) tumors, the ICC was very low and this may be a consequence of the small number of positive answers obtained for these questions. An additional reason that may partially explain some of the low ICC values is that upon retrieval of confirmatory documents during GCRA, some of the cancer diagnoses were not confirmed. This occurred particularly often in the multiple primary cases, and in those, OC was usually not confirmed. Uncertainty about the cancer family history, especially when there is more than one cancer diagnosis in the family, may also be frequent at the first visit in the primary care unit and may be confirmed or refuted before the genetic evaluation. Another concern would be a potential bias of ascertainment due to non-inclusion of women who did not attend genetic cancer risk assessment sessions. In fact, the demographic data of women with a positive family history who underwent GCRA (attenders) differed significantly in some aspects from those of women who did not undergo GCRA (non-attenders). Non-attenders were generally younger, less educated, and had undergone a breast biopsy less often than attenders (p < 0.001), as demonstrated elsewhere. However, for most of the questions in the family history questionnaire, there was no significant difference between the proportion of positive answers between attenders and non-attenders [27].

Regarding the ability of FHS-7 to identify particular HBC phenotypes, the presence of at least one positive answer was associated with good sensitivity at acceptable specificity. This was particularly the case for the identification of the HBOC phenotype, and less for the Li-Fraumeni-Like phenotype, however, the instrument was not originally developed to identify Li-Fraumeni-Like families. The worst performance was in the identification of Li-Fraumeni-Like families and all of them were defined as Li-Fraumeni-Like by Eeles criteria only [42]. These findings indicate that individuals with family history features suggesting Li-Fraumeni syndrome variants, especially those fulfilling the more relaxed criteria of Eeles may be missed by the instrument. Since the major goal of using the instrument was to correctly identify HBC phenotypes in general, the very high negative predictive values obtained considering cut point 1 was highly satisfactory, even if this required evaluation of a larger number of individuals to ensure that only a very small fraction of high-risk cases remain unidentified. The relatively low specificity of FHS-7 must be considered as a potential limitation for this study. However, if one considers the context of the study, including the low cost involved in the application of the instrument as well as of clinical genetic evaluations, and the added significant benefit of identification of a true hereditary breast cancer family, in which several at-risk individuals may benefit from further genetic testing and screening interventions, the low specificity becomes less important. An additional limitation of the study is that we have not considered limited family structure in the pedigree analyses. Weitzel *et al*. (2007) [43] have recently described an increased prevalence of germline mutations in women diagnosed with early-onset breast cancer, who may not fulfill well-established criteria for a hereditary breast cancer syndrome because of the lack of females in either lineage. If this would have been considered, prevalence of phenotype-positive cases would have likely increased.

A significant proportion of women from the community-based sample studied here presented a family history of BC, OC and/or CRC and upon genetic risk evaluations: 6.2% of them had phenotypes of specific BC predisposition syndromes. These results have been reported elsewhere in detail in [27]. Even considering that the majority of families with family histories of a hereditary breast cancer syndrome fulfilled the more relaxed criteria for Li-Fraumeni-like syndrome, the identification of such families may still be relevant since this geographic has been identified as a region with high prevalence of a specific *TP53 *germline mutation.

## Conclusion

In conclusion, simple family history questionnaires such as the one developed here can be used in BC risk-screening programs at the primary care level as important tools for the identification of individuals who may benefit from specific interventions.

## Competing interests

The authors declare that they have no competing interests.

## Authors' contributions

PAP, EIP, LK and MC developed the initial design of the genetics study within the cohort. JG, FLR, EA, SMM, EB and VBR contributed to data acquisition and handling. PAP, JG, AVS and SC were responsible for data analysis, and interpretation. All authors revised the manuscript and agreed with the final submitted version of the manuscript.

## Pre-publication history

The pre-publication history for this paper can be accessed here:

http://www.biomedcentral.com/1471-2407/9/283/prepub
